# Estimating and Projecting Efficacy of Monoclonal Antibodies Against Respiratory Syncytial Virus Beyond Clinical Trial Periods

**DOI:** 10.3390/vaccines14090737

**Published:** 2026-08-26

**Authors:** Dawei Wang, John C. Lang, Klodeta Kura, Yao-Hsuan Chen

**Affiliations:** 1 Health Economic and Decision Sciences, Merck & Co., Inc., Rahway, NJ 07065, USA; 2 Health Economic and Decision Sciences, Merck Canada Inc., Kirkland, QC H9H 4M7, Canada; 3 Health Economic and Decision Sciences, MSD (UK) Limited, Moorgate, London EC2M 6UR, UK

**Keywords:** respiratory syncytial virus, monoclonal antibodies, nirsevimab, clesrovimab, waning efficacy, Bayesian inference, Gaussian process, incidence rate ratio

## Abstract

Background: Clinical trials of respiratory syncytial virus (RSV) monoclonal antibodies (mAbs) have followed participants for up to 180 days, covering a typical single RSV season. However, to assess the full clinical impact of mAb administration, post-trial efficacy estimates are needed, particularly in countries where RSV circulation extends beyond six months. Methods: In this study, we applied a previously published Bayesian framework to estimate how the level of protective efficacy against RSV provided by clesrovimab and nirsevimab changes over the first year after administration. Using this Bayesian framework, which modeled new infections as Poisson processes with a Gaussian process prior for the force of infection and a parametric waning function for the intervention arm, we estimated placebo incidence rates and posterior waning functions. We validated our estimates by calculating model-estimated cumulative trial-period efficacy using an Incidence Rate Ratio (IRR) approach and by reconstructing event-free probability curves, comparing both with published clinical trial results. Results: Model-derived cumulative efficacy estimates closely matched trial-reported results for both products. The model-projected instantaneous probability of being protected at one year—an extrapolation beyond the observed trial follow-up—was 24.6% (95% CrI: 1.6–57.0%) for clesrovimab and 12.8% (95% CrI: 1.2–44.4%) for nirsevimab, with substantially overlapping credible intervals. Conclusions: Both RSV mAbs were projected to provide some level of protection throughout the first year following administration, with broadly similar levels of protection.

## 1. Introduction

Respiratory Syncytial Virus (RSV) remains a leading cause of lower respiratory tract infections (LRTI) in infants worldwide [1]. Unlike traditional active vaccines, long-acting monoclonal antibodies (mAbs) such as nirsevimab and clesrovimab provide immediate, passive immunization specifically designed to protect infants through their first RSV season [2,3]. However, because the duration of protection is finite, understanding the waning efficacy of mAbs, i.e., the longitudinal profile of mAb protection, is critical for determining clinical dosing schedules and assessing public health impact in the context of seasonal RSV epidemics [4].

Randomized controlled trials (RCTs) typically report a single, cumulative efficacy estimate over a fixed follow-up period [2,3]. However, protection from treatments is inherently dynamic. For example, a mAb may provide near-complete protection during the initial months after administration, followed by a gradual decline as antibody titers fall below the protective threshold, e.g., as assumed in the modeling approach of Hodgson et al. (2024) [4]. While “seasonal” or “seasonal with catch-up” administration strategies are considered optimal in many high-income countries with temperate climates and predictable winter RSV peaks [5,6], many regions—particularly in tropical and subtropical climates—experience year-round RSV circulation, and are therefore considering “year-round” administration strategies [7]. In this context, the efficacy profile of the mAb immunoprophylaxis over a year-long (365-day) horizon is a primary determinant of clinical impact [4]. Similarly, in a seasonal context, the efficacy profile remains important, as not all RSV cases occur during the RSV season; for example, in China nearly 30% of RSV-positive tests occur between April and September [8]. Quantifying the waning profile of mAbs is therefore essential for robust cost-effectiveness analyses in both seasonal and year-round setting. Notably, to date all countries that have introduced RSV mAb immunoprophylaxis have adopted a seasonal (or seasonal-with-catch-up) strategy; no country—including those with year-round RSV circulation—has yet recommended year-round administration. Because such a strategy has not been deployed anywhere, no real-world data on its performance can exist, and a model that characterizes protection across the full 365-day horizon is required to evaluate a year-round versus seasonal strategy prospectively.

The objective of this study was to validate and extend a previously published Bayesian “infection pressure” framework to estimate the time-varying degree of protection of nirsevimab and clesrovimab against various medically attended RSV endpoints over a 1-year (365-day) period. By decoupling mAb waning efficacy from trial-specific seasonal RSV incidence, this study aimed to estimate waning profiles for nirsevimab and clesrovimab that could be applied to assess the clinical and economic impact of RSV mAb immunoprophylaxis across diverse seasonal or year-round contexts.

## 2. Materials and Methods

### 2.1. A Bayesian Framework for Estimating Waning Efficacy

Standard clinical trial reporting summarizes intervention efficacy as a single cumulative value over the follow-up period, typically within a frequentist framework based on incidence density. For example, an Incidence Rate Ratio (IRR) is calculated by comparing infections per person-time at risk in the treatment with that in the placebo arm [2,3]. While this cumulative value is appropriate for trial reporting, it does not describe how protection changes over time; that is, it does not characterize the waning profile (i.e., the degree of protection as a function of time) either during or beyond the trial period. Estimating this time-varying degree of protection is a distinct goal that can be pursued using either a frequentist or a Bayesian approach. We adopted the Bayesian framework of Hodgson et al. (2024) [4] because it performs well when evidence is sparse, as is the case for efficacy beyond the trial follow-up period, and because it more fully quantifies the uncertainty around the estimated waning profile.

The Bayesian approach developed by Hodgson et al. (2024) [4] was applied to RCT data to estimate the waning protective efficacy of nirsevimab both during and beyond the 5-month RCT follow-up period. In the study, new medically attended RSV outcomes were modeled as events in a non-homogeneous Poisson process. For the control (no intervention) arm, the force of infection was modeled as a Gaussian Process (GP), i.e., a flexible non-parametric function capable of reproducing the observed seasonality in the force of infection. For the mAb intervention arm, the force of infection was assumed to be the product of the natural force of infection multiplied by the complement of a monotonically decreasing parametric waning function. For example, following Hodgson et al. (2024) [4], in the base case analysis waning function was assumed to be proportional to the cumulative distribution function of an Erlang-3 random variable. In this context, the value of the waning function at a specific time was interpreted as the degree of protection, or the instantaneous probability of being protected from infection. Throughout, we use “degree of protection” to denote the instantaneous probability of being protected at a given time since administration, i.e., the value of the waning function at that time, and “waning profile” to denote the trajectory of the degree of protection over time. Cumulative efficacy, by contrast, refers to the IRR-based summary of protection over a fixed follow-up window, as reported by clinical trials.

### 2.2. Data

As a verification exercise, we reproduced the analysis of Hodgson et al. (2024) [4] by fitting the above model to the same data set they used to estimate the 1-year waning efficacy profile of nirsevimab. In particular, the model was fitted with the digitized Kaplan-Meier (KM) curve from the pooled results of the MELODY/Phase 2b/3 RCT for the MALRI endpoint [2]. To extend this approach to clesrovimab, we also estimated its 1-year waning efficacy profile against MALRI. To align efficacy endpoints between nirsevimab and clesrovimab trials, the model was calibrated to the KM curve from a post-hoc analysis of clesrovimab CLEVER RCT data using the endpoint of medically attended lower respiratory infection (LRI) requiring ≥ 2 indicators of LRI/severity [3]. We note that the primary efficacy endpoint for clesrovimab was RSV-associated MALRI that resulted in at least one indicator of LRI or disease severity from days 1 to 150 after injection. Published Kaplan–Meier curves were digitized using WebPlotDigitizer (version 5.2) [9], extracting the event-free probability coordinates for each trial arm; the corresponding event data used for model calibration were then reconstructed from these coordinates. The accuracy of the digitization was verified by comparing the KM-derived cumulative efficacy with the trial-reported estimates.

### 2.3. Model Estimation

The model was calibrated by maximizing a posterior function defined as the product of the likelihood functions for the no-intervention and intervention trial arms and the prior distribution of the model parameters and the GP. Because the posterior distribution was analytically intractable, it was estimated using Markov Chain Monte Carlo (MCMC) methods [10]. To ensure a thorough exploration of the posterior distribution, 10 MCMC chains were run in parallel. Convergence of the chains was assessed using the potential scale reduction factor, $\hat{R}$, which compared the between-chain and within-chain variances to determine whether all chains were well mixed. Specifically, values of $\hat{R}<1.1$) were taken to indicate convergence.

### 2.4. Model Validation

Calibrated models for the nirsevimab and clesrovimab MALRI endpoints were validated by comparing model outputs with RCT data using two approaches. First, survival curves for the control (no-intervention) and mAb (intervention) arms were estimated from the calibrated model using a recursive susceptible-depletion algorithm. These model-based survival curves were then compared with the original KM curves to validate the internal consistency of the modeling framework. Second, a recursive IRR approach was applied to the calibrated model to estimate cumulative efficacy over the trial period. The resulting model-based estimates were compared with trial-based estimates to confirm that this modeling approach preserved the cumulative efficacy.

### 2.5. Scenario Analysis

Scenario analyses were conducted to assess the sensitivity of the model to two structural choices. First, with respect to the base case choice of a GP to model the force of infection, the model was re-calibrated using random-walk penalized B-spline (RW Spline) as the non-parametric estimator for the force of infection. Second, with respect to the base case choice of Erlang-3 cumulative distribution function for the parametric waning function, a total of seven different functional forms were considered for the waning function (see Table 1). Throughout, *a* denotes the initial efficacy, *b* the primary waning rate or scale parameter, and the remaining symbol (*k*, η, or σ) the form-specific shape parameter, as listed in Table 1; this notation is used consistently hereafter. In total, for each intervention-endpoint combination, fourteen models were calibrated by combining two non-parametric approaches for the force of infection with seven parametric approaches for the waning efficacy function. The “goodness of fit” among those fourteen calibrated candidate models was evaluated using the Expected Log Pointwise Predictive Density (ELPD), a common model selection criteria for evaluating out-of-sample predictive performance [11]. In addition to the ELPD, the leave-one-out (LOO) penalty ($p_{\mathrm{LOO}}$) was evaluated as a proxy for effective model complexity to ensure that the complexity of the non-parametric GP and RW Spline remained available data density, that is, the amount of data available for model calibration [11].

## 3. Results

### 3.1. Model Estimation

Convergence was achieved for all models, with 100% MCMC chains achieving $\hat{R}<1.1$. For example, for the base case Erlang-3 specification, the effective number of parameters ($p_{loo}$) was 5.5 for nirsevimab and 5.2 for clesrovimab, consistent with the nominal parameter counts (Table 2).

**Figure 1:**
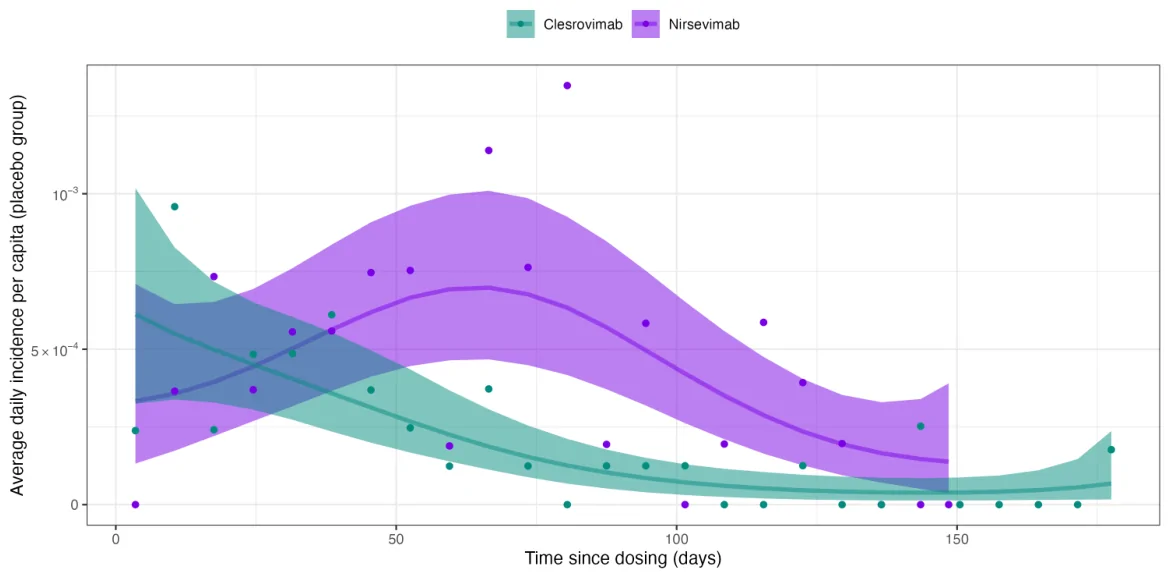
One-week moving average of daily per capita incidence (placebo group) for nirsevimab and clesrovimab clinical trials. Dots represent clinical trial data; solid lines and shaded areas represent model estimates and 95% credible intervals, respectively.

The Bayesian framework successfully captured the underlying epidemiological dynamics of the respective clinical trial periods. Under the base case (GP and Erlang 3) model specification, Figure 1 shows the one-week moving average of daily per capita incidence in the placebo cohorts from both trials alongside the corresponding model-derived estimates. The model closely tracked the distinct seasonal patterns observed in the trials: while the nirsevimab trials generally began before the RSV seasonal peak (resulting in a relatively symmetric distribution of infection pressure), the clesrovimab trial experienced immediate peak pressure followed by an extended observational tail, reflecting the challenge of coordinating trial timing with the RSV season temporarily disrupted by the COVID-19 pandemic.

**Figure 2:**
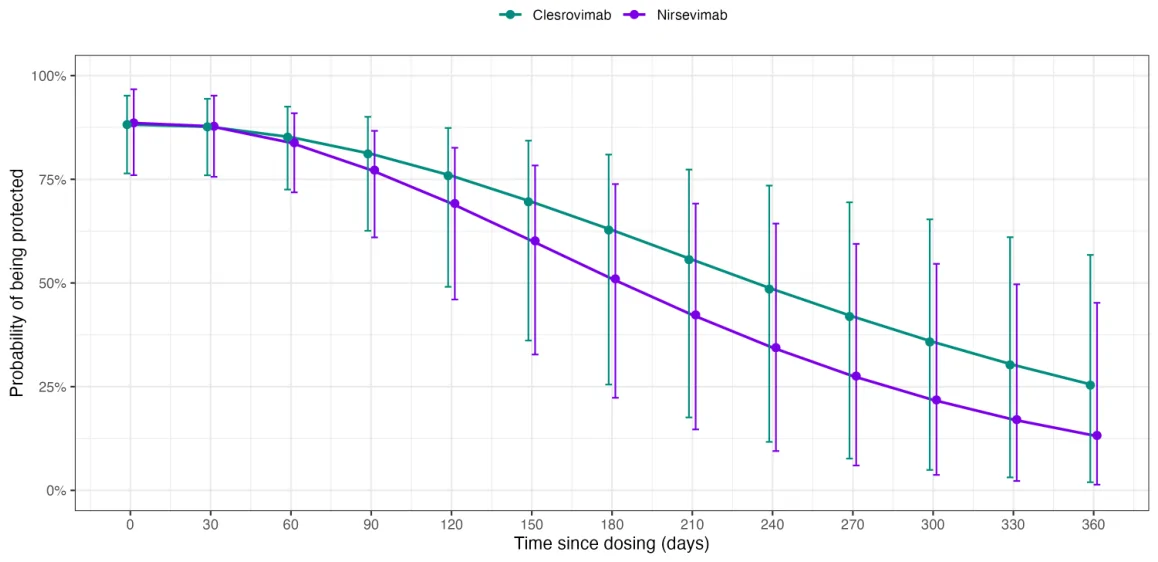
Instantaneous probability of protection (i.e., degree of protection) against medically attended lower respiratory infection as a function of time since administration, under the base-case model (Gaussian-process force of infection with Erlang-3 waning function). Dots and lines show posterior medians for clesrovimab and nirsevimab; bars show 95% credible intervals. Trial follow-up extended to approximately Day 150 for nirsevimab and Day 180 for clesrovimab.

Under the base case model specification, the posterior trajectories of the instantaneous probability of protection (i.e., the degree of protection) revealed comparable waning profiles for both monoclonal antibodies (Figure 2). The geometric median for the fitted scale parameter (*a*) was similar between products (clesrovimab: 0.88; nirsevimab: 0.88), while the rate parameter (*b*) suggested a slightly more sustained profile for clesrovimab (clesrovimab: 101.6; nirsevimab: 81.5). By the end of the 365-day horizon, the instantaneous probability of protection was estimated at 24.6% (95% CrI: 1.6–57.0%) for clesrovimab and 12.8% (95% CrI: 1.2–44.4%) for nirsevimab.

### 3.2. Model Validation

For the base case model (GP and Erlang-3), the model-based 150 day post-dose efficacy was estimated at 86.5% (95% CrI: 75.3–93.2%), consistent with the trial-reported efficacy of 88.0% [3] as shown in Table 2. This level of agreement was maintained over the extended 180-day window, where the model estimated efficacy at 85.7% (95% CrI: 74.3–92.6%), compared with the trial-reported 87.2%. A similar degree of alignment was observed for nirsevimab, further validating the framework’s ability to accurately reconstruct trial dynamics across RSV mAb products and clinical datasets.

**Figure 3:**
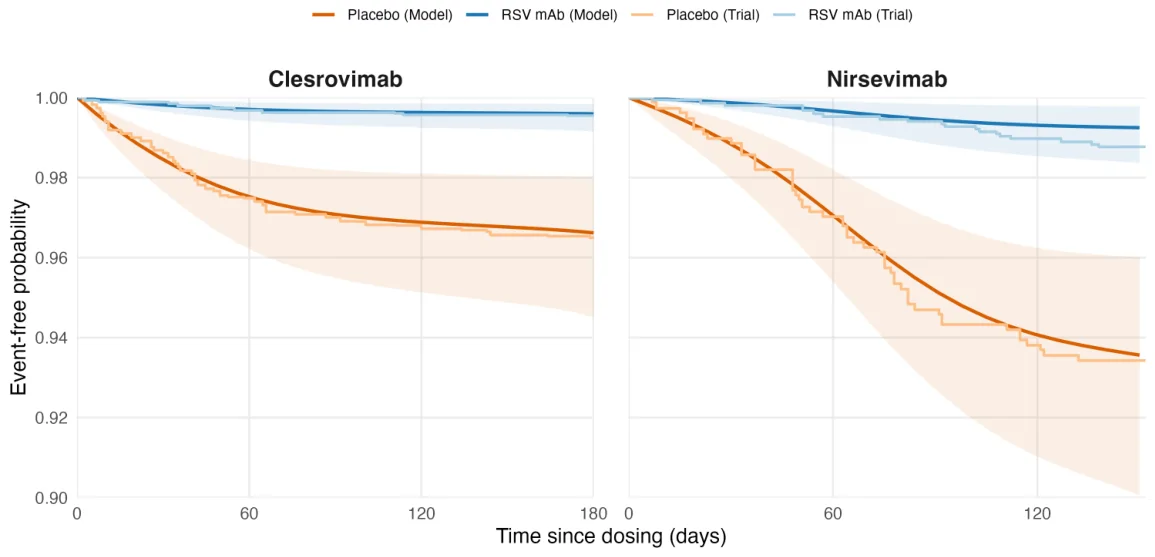
Model-based estimated event-free probability curves vs. digitized Kaplan-Meier plots from clinical trial data. Solid lines and shaded areas represent model estimates and 95% credible intervals, respectively.

Further validation was provided through the reconstruction of event-free probability curves. The ErLang-3 model-based survival trajectories demonstrated a strong visual and statistical agreement with KM curves (Figure 3). This longitudinal alignment confirmed that the recursive susceptible depletion algorithm correctly integrated the time-varying force of infection with treatment-specific waning functions, providing a robust foundation for long-term projections.

### 3.3. Scenario Analysis

For nirsevimab, the Erlang-3 and Gompertz architectures demonstrated the highest predictive accuracy, with the Erlang-3 model providing the best balance between fit and complexity (ELPD=−67.7). The model-derived cumulative efficacy at Day 150 (81.7%; 95% CrI: 70.1–89.3%) closely matched the efficacy estimated from the digitized KM plot of 81.9%. In contrast, the Exponential model performed the worst (ELPD=−70.8) and produced a markedly lower efficacy estimate (67.7%), failing to replicate the sustained clinical protection reported in the MELODY trial. Long-term projections to Day 365 exhibited substantial differences across waning assumptions; while the Erlang-10 model suggested relatively durable protection (63.1%), the Erlang-3 model predicted a more conservative late-season decay (12.8%).

For clesrovimab, the Erlang-10 and Erlang-3 architectures were the top-performing models (ELPD of −57.1 and −57.5, respectively). The Day 180 efficacy estimate from the Erlang-3 base case (85.7%; 95% CrI: 74.3–92.6%) aligned closely with the trial-reported 87.2% (95% CI: 75.1–93.4%). While the Sigmoid model also provided strong short-term agreement (86.6% at Day 180), it predicted an absolute loss of protection by Day 365 (0.0%).

The results presented above, which used GP non-parametric form to model the force of infection in the no-intervention arm, were identical with results for the RW Spline. These scenario findings indicated that the base-case results are robust to the specific choice of non-parametric approach used to model background force of infection.

## 4. Discussion

The ELPD differences among the top-performing models are small: for clesrovimab, Erlang-10 versus Erlang-3 differ by approximately 0.4 ELPD points, and for nirsevimab, Gompertz versus Erlang-3 by approximately 0.2 points (Table 2). Differences of this magnitude are well within the expected uncertainty of out-of-sample model comparison [11], and the trial-period data therefore cannot meaningfully discriminate among these specifications. Given this statistical near-equivalence, the Erlang-3 form was selected as the common base case on transparent secondary criteria: consistent performance across both products, parsimony, continuity with the published base case of Hodgson et al. (2024) [4], and avoidance of specifications whose apparent advantage depends on late-stage behavior that is unconstrained by the data. The alternative forms are retained as structural scenario analyses.

While both mAbs exhibit similar initial performance, the 12-month projections reveal critical insights into their long-term clinical durability. Both clesrovimab and nirsevimab are projected to maintain some level of protection throughout the first year following administration. Overall, our analysis indicates that these two immunoprophylaxis options offer broadly similar levels of protection at the one-year horizon. Given the overlapping 95% credible intervals, these results should not be interpreted as demonstrating superiority of either agent.

An important finding of the scenario analysis is that several mathematically plausible waning functions fit the observed trial period comparably well—with similar ELPD values and near-identical reconstruction of trial-period cumulative efficacy—while producing markedly different projections at Day 365 (for clesrovimab, from 0.0% under the Sigmoid form to 78.8% under Erlang-10; for nirsevimab, from 0.0% to 63.1%). Even under the base-case Erlang-3 specification, the 95% credible intervals at Day 365 span from near-zero protection to over 40–50%. Data limited to 150–180 days of follow-up cannot discriminate among these long-term behaviors, and the Day 365 point estimates should therefore be interpreted in light of this substantial structural uncertainty. For downstream health-economic applications, parameter uncertainty in the fitted waning curve can be propagated into probabilistic sensitivity analysis using the full posterior distribution, whereas the divergence across candidate waning functions is best handled as structural scenario analyses.

The Bayesian framework applied by Hodgson et al. (2024) [4] decouples treatment effects from seasonal background noise. Our analysis confirms that the core architecture—utilizing a latent Gaussian Process to model infection pressure alongside a parametric waning function—is robust and capable of capturing the fundamental kinetics of monoclonal antibody (mAb) protection. However, we identify a critical limitation in how cumulative efficacy is traditionally calculated from these posterior distributions. Specifically, Hodgson et al. (2024) [4] approximated cumulative efficacy using the arithmetic mean of the waning function over the follow-up period rather than a recursive incidence-rate-ratio (IRR)-based approach. An unweighted average implicitly treats every day of follow-up as contributing equally, regardless of how much RSV is actually circulating; under a seasonal force of infection this approach may over- or under-estimate cumulative efficacy depending on the timing of protection relative to periods of high transmission. In this work, we therefore recover cumulative efficacy using a recursive, IRR-based approach that weights protection by the time-varying force of infection, reproducing the standard estimand reported in clinical trials.

A recent non-peer-reviewed preprint posted on medRxiv by Gong et al. (2025) [12], a follow-up to Hodgson et al. (2024) [4], applied the same framework to clesrovimab and nirsevimab and reached a broadly consistent conclusion that the two monoclonal antibodies confer comparable protection. A few technical differences between the two analyses are worth noting. Their main-text head-to-head comparison used the clesrovimab primary endpoint, which is not aligned with the nirsevimab case definition. Hospitalization outcomes were likewise misaligned (“first hospital admission for medically attended RSV LRTI” for nirsevimab versus “RSV-associated hospitalization” for clesrovimab) and, because the framework requires a digitized Kaplan–Meier curve as input, the aligned clesrovimab endpoint (“hospitalization for LRI”) cannot be used, as no such curve has been published. In addition, their preprint code fitted the candidate waning forms jointly within a single model rather than independently—an implementation detail corrected in a subsequent code revision, but not in the preprint version—though this has negligible effect on the estimated curves. Our analysis prioritized endpoint alignment across products and considered a broader set of parametric waning forms together with alternative non-parametric estimators for the background force of infection.

The primary objective of this work was to estimate the time-varying degree of protection of nirsevimab and clesrovimab. The framework’s validity is supported by the close agreement between our estimates with reported trial data: for nirsevimab, the 150-day estimate (81.2%) aligned with the MELODY trial’s frequentist efficacy (79.5%) and the static IRR derived from the Kaplan-Meier (KM) plot (81.9%). Similarly, for clesrovimab, our 150-day estimate (85.8%) and 180-day estimate (84.9%) demonstrated strong agreement with the CLEVER trial’s reported efficacy and KM-derived IRR values of 88.0% and 87.2%, respectively.

Beyond statistical metrics like ELPD, the selection of the Erlang-3 architecture was also driven by its ability to perform consistently across products. While certain alternative models, such as Erlang-10 or Log-normal distribution, provided marginally better fits for individual cohorts, the Erlang-3 was the only functional form that demonstrated robust performance for both nirsevimab and clesrovimab. This broader consistency suggests that the Erlang-3 form provides a pragmatic common benchmark for characterizing waning within this class of immunoprophylaxis, although this reflects statistical model selection rather than direct biological evidence about the mechanism of antibody decay. Choosing a consensus model rather than selecting the top-performing kernel for each individual trial reduces the risk of over-fitting and provides a more standardized benchmark. By prioritizing a model specification that works across the entire class of immunoprophylaxis, we move away from idiosyncratic projections and toward a more reliable, platform-based understanding of mAb durability.

These projections are relevant for policy even as real-world evidence accumulates. Trials and real-world effectiveness studies report a single cumulative estimate over a fixed window, whereas cost-effectiveness and dynamic transmission models require the continuous, time-varying degree of protection; by decoupling intrinsic waning from the seasonal force of infection, the estimated profiles are transferable across the temperate, tropical, and year-round settings where these products are used. Because no country has yet adopted year-round dosing, the year-round-versus-seasonal comparison can only be examined prospectively through modeling. The model is complementary to real-world data—which can be used to validate and update these profiles as longer-duration evidence matures—and is anchored to the observed trials, reproducing reported cumulative efficacy.

In most temperate settings the RSV season spans approximately six months and current immunization programs are seasonal; protection over the trial-observed window is therefore the primary driver of clinical benefit in those settings. The full-year waning profile nevertheless remains relevant for three reasons. First, many tropical and subtropical regions experience extended or year-round RSV circulation and are considering year-round administration strategies, which—since no country has yet implemented one—can only be evaluated prospectively through modeling. Second, even in seasonal settings a non-negligible share of RSV cases occurs outside the core season, and the timing of administration relative to season onset varies between infants. Third, cost-effectiveness and transmission models require the continuous, time-varying degree of protection as an input, rather than a single fixed-window cumulative estimate.

### 4.1. Limitations

The primary limitation of this study lies in the structural assumptions underlying the parametric waning functions, which may not fully capture the complexity of long-term pharmacokinetic-pharmacodynamic (PK/PD) interactions beyond the clinical follow-up period. While the Erlang-3 architecture successfully reconstructed trial dynamics by capturing the initial stability and subsequent decay of protection, the extrapolation to a full year remains a model-based projection that lacks direct longitudinal validation from extended-duration clinical data. Despite this constraint, the high degree of agreement between our recursive estimates and published frequentist results suggests that this framework provides a robust and biologically plausible foundation for evaluating the comparative cost-effectiveness of mAbs across diverse seasonal conditions.

A further limitation concerns endpoint alignment. Because the primary clesrovimab endpoint (RSV-associated MALRI with at least one indicator of LRI or disease severity, days 1 to 150) differs from the nirsevimab MALRI case definition, the head-to-head comparison relied on a post-hoc clesrovimab endpoint (medically attended LRI) selected to align the two case definitions. Reliance on a post-hoc endpoint, and on the availability of a digitized Kaplan-Meier curve for that endpoint, may introduce residual differences that are not attributable to the products themselves. More generally, no head-to-head randomized or real-world comparisons of clesrovimab and nirsevimab currently exist. The estimates presented here derive from separate randomized trials that differ in patient populations, epidemiological context (including the COVID-19–disrupted RSV seasonality during the CLEVER trial), study design, and endpoint construction, and the endpoint harmonization relied on a post-hoc clesrovimab endpoint. The analysis therefore constitutes an indirect comparison of waning profiles estimated from separate trials and should not be interpreted as a comparative-effectiveness assessment.

### 4.2. Conclusions

Using a Bayesian infection-pressure framework, we estimated the time-varying degree of protection of nirsevimab and clesrovimab over a one-year horizon. Both monoclonal antibodies were projected to provide some level of protection throughout the first year following administration, with broadly similar levels of protection (instantaneous probability of protection at day 365: 24.6% [95% CrI: 1.6–57.0%] for clesrovimab and 12.8% [95% CrI: 1.2–44.4%] for nirsevimab). The waning profiles were well described by an Erlang-3 function, with a similar geometric-median scale parameter for both products (approximately 0.88) and rate parameters of 101.6 for clesrovimab and 81.5 for nirsevimab. These estimates were validated against trial-reported cumulative efficacy using a recursive IRR approach and against digitized Kaplan-Meier event-free probability curves from both trials. A single parametric architecture (Erlang-3) accurately characterized the waning kinetics of both antibodies across diverse seasonal contexts, and by formalizing model selection through information criteria (ELPD) and assessing structural uncertainty at the 12-month horizon, we establish a protocol for more transparent longitudinal projections.

## Figures and Tables

**Table 1:** Parametric Functional Forms Evaluated for Monoclonal Antibody Waning Efficacy.

Model Name	Description	Parameters
Exponential	Assumes a constant rate of waning over time; the simplest decay model.	a,b
Erlang-3	Protection modeled through three stages (k=3); creates an initial plateau (shoulder) of efficacy.	a,b,k=3
Erlang-10	Higher-order plateau (k=10) simulating a very stable antibody followed by rapid late-stage waning.	a,b,k=10
Gompertz	A flexible model where the waning rate itself changes exponentially over time.	a,b,η
Log-normal	Assumes protection duration follows a log-normal distribution; allows for a rapid peak and long tail.	a,b,σ
Weibull	Versatile distribution capable of representing increasing, decreasing, or constant waning rates.	a,b,k
Sigmoid	Represents a sharp transition from high to low efficacy, characterized by a symmetric S-shaped curve.	a,b,k

Note: *a* initial efficacy; *b* denotes the primary waning rate or scale parameter.

**Table 2:** Validation and sensitivity analysis of the clesrovimab and nirsevimab waning profiles across parametric waning functions.

Model	KM-Derived Efficacy (150 Days)	Model-Estimated Efficacy (150 Days)	KM-Derived Efficacy (180 Days)	Model-Estimated Efficacy (180 Days)	Degree of Protection Day 365	ELPD	ΔELPD	$p_{\mathrm{loo}}$
*Clesrovimab*								
Exponential	88.0%	75.3% (61.0, 83.8)	87.2%	73.8% (59.4, 82.5)	7.6% (1.3, 18.0)	−61.67	4.55	5.42
**Erlang-3**	**88.0%**	**86.5%** **(75.3, 93.2)**	**87.2%**	**85.7%** **(74.3, 92.6)**	**24.6%** **(1.6, 57.0)**	**−57.55**	**0.43**	**5.20**
Erlang-10	88.0%	87.0% (75.6, 93.6)	87.2%	87.0% (75.6, 93.6)	78.8% (1.1, 91.7)	−57.12	0	4.90
Gompertz	88.0%	86.4% (74.6, 93.3)	87.2%	85.6% (73.5, 92.8)	0.1% (0.0, 83.1)	−57.98	0.86	5.63
Log-normal	88.0%	86.8% (75.1, 93.5)	87.2%	86.5% (74.6, 93.2)	59.1% (0.7, 90.6)	−57.41	0.29	5.20
Weibull	88.0%	86.1% (73.1, 93.1)	87.2%	85.6% (72.4, 92.8)	54.6% (0.2, 88.0)	−57.71	0.59	5.31
Sigmoid	88.0%	86.9% (75.4, 93.5)	87.2%	86.6% (74.9, 93.3)	0.0% (0.0, 0.0)	−57.43	0.31	5.31
*Nirsevimab*								
Exponential	81.9%	67.7% (54.1, 76.5)	—	—	10.1% (2.9, 19.5)	−70.78	3.05	5.25
**Erlang-3**	**81.9%**	**81.7%** **(70.1, 89.3)**	**—**	**—**	**12.8%** **(1.2, 44.4)**	**−67.73**	**0**	**5.46**
Erlang-10	81.9%	81.7% (69.2, 89.5)	—	—	63.1% (0.0, 86.8)	−68.98	1.25	5.89
Gompertz	81.9%	80.7% (67.7, 88.6)	—	—	0.0% (0.0, 48.5)	−67.92	0.19	5.84
Log-normal	81.9%	81.5% (69.5, 89.3)	—	—	26.5% (0.0, 82.9)	−68.39	0.66	5.94
Weibull	81.9%	80.6% (67.4, 88.7)	—	—	31.2% (0.0, 78.2)	−68.25	0.52	5.63
Sigmoid	81.9%	81.2% (68.6, 89.2)	—	—	0.0% (0.0, 0.0)	−69.52	1.79	6.30

Values in parentheses are 95% credible intervals. KM-derived efficacy is estimated from the digitized Kaplan–Meier (KM) input data, whereas model-estimated efficacy is the cumulative incidence-rate-ratio from the calibrated model. The best-fitting base-case model (Erlang-3) is shown in bold. ΔELPD, difference in ELPD relative to the best-performing model for each product [11]. Abbreviations: KM, Kaplan–Meier; ELPD, expected log pointwise predictive density; $p_{\mathrm{loo}}$, leave-one-out effective number of parameters; —, not available (the nirsevimab MELODY follow-up did not extend to 180 days).

## Data Availability

The digitized data and code supporting the reported results are available from the corresponding author upon reasonable request.
